# Transforming a transcription factor

**DOI:** 10.7554/eLife.33792

**Published:** 2018-01-08

**Authors:** Robert D Burke

**Affiliations:** Department of Biochemistry and MicrobiologyUniversity of VictoriaVictoriaCanada

**Keywords:** sea urchins, Alx1 transcription factor, skeletogenesis, gene duplication, exon extension, evolution, Other

## Abstract

A transcription factor that regulates skeleton formation in sea urchin embryos has evolved a new domain that is essential for this process.

**Related research article** Khor JM, Ettensohn CA. 2017. Functional divergence of paralogous transcription factors supported the evolution of biomineralization in echinoderms. *eLife* **6**:e32728. doi: 10.7554/eLife.32728

As an embryo develops, complex regulatory networks control where and when genes are activated, resulting in tissues and organs forming at the right time and place. Changes to these networks, in particular to DNA sequences that bind transcription factors, can affect how an organism develops and looks (Carroll, 2008; Wray, 2007; Peter and Davidson, 2011).

Transcription factors are proteins that help turn specific genes on or off by binding to nearby DNA, and a somewhat controversial theory suggests that changes affecting the strength of transcription factor binding may modify regulatory networks (Lynch and Wagner, 2008). Such changes are, however, less favored, as they could affect many genes and thus have a negative impact on the fitness of an organism. Now, in eLife, Jian Ming Khor and Charles Ettensohn of Carnegie Mellon University report how a specific region on a transcription factor can indeed affect skeleton formation in sea urchins (Khor and Ettensohn, 2017).

Sea urchins are a popular model organism in developmental biology and like most other echinoderms, the larvae of sea urchins are very different to their adult form. Sea urchins develop from an egg into a planktonic larva, before transforming into a bottom-dwelling juvenile, and unlike most echinoderms, they form a larval skeleton in the early embryonic stages (McClay, 2011).

A well-studied group of cells called the micromeres are key to this process, and are the first cells internalized as the embryo acquires its form (McIntyre et al., 2014). The molecular mechanisms that determine their fate have thus received considerable attention and were one of the first examples of a developmental gene regulatory network (Davidson et al., 2002; Ettensohn, 2009). Some of the molecular components of this network are located in the unfertilized egg and are divided unequally between new cells. Within the micromeres, regulatory genes control a hierarchical network of genes, which causes them to build a precisely positioned and patterned skeleton (Figure 1A–D).

**Figure 1.**
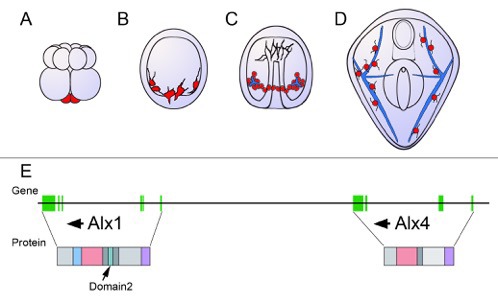
Schematic showing the development of the sea urchin from embryo to larva. (**A**) During the 16-cell stage, a cluster of four small cells called the micromeres (red) form at one pole of the embryo. (**B**) The micromeres then develop into primary mesenchyme cells (also shown in red) and migrate into the embryo. (**C**) As the embryo turns into a hollow ball of cells, the primary mesenchyme assembles into a ring with two clusters of cells (red), positioned ventrally. During this phase, the cells fuse and begin to secrete a calcium carbonate skeleton (shown in blue). (**D**) In the larva, the skeleton has developed into a pair of skeletal rods (blue) that grow to support and shape the larva. (**E**) The genes Alx1 and Alx4 are adjacent and are thought to have arisen from a duplication early during the evolution of echinoderms. Arrows indicate the orientation of the genes. The genes are similar, but differ in the regions of the gene that encode the protein sequence (vertical green bars). The proteins also have several identical domains, but Domain 2 (shown in turquoise), which is critical to skeleton formation, is only found in Alx1. Data for the gene organization of the purple sea urchin *Strongylocentrotus purpuratus* (as displayed here) has been obtained from the echinoderm genomic database ‘Echinobase’.

One key transcription factor in this regulatory network is Alx1, which is exclusively activated or expressed in the micromeres soon after they form (Ettensohn et al., 2003). Previous research has shown that when Alx1 is blocked, the embryos of sea urchins develop without forming a skeleton, but when Alx1 is overexpressed in cells other than the micromeres, they develop into skeleton-forming cells.

Khor and Ettensohn blocked Alx1 with a compound called a morpholino, and at the same time, injected the embryo with a version of Alx1 that is insensitive to this substance and restores skeleton formation. To identify the roles of the Alx1 protein, they deleted or mutated various parts of the morpholino-insensitive Alx1 and tested if the embryos were still able to build a skeleton. Most parts of the protein were dispensable, but a small domain unique to Alx1, called Domain 2, turned out to be essential for skeleton formation. Furthermore, Khor and Ettensohn discovered that when Domain 2 was inserted into Alx4, which is an adjacent copy of Alx1, sea urchins were able to form a normal skeleton.

Khor and Ettensohn then compared the genomes of other echinoderms and discovered that Alx4 was highly conserved within all members of this group, whereas Alx1 varied greatly. The Alx1 proteins of close relatives of the sea urchin were functionally interchangeable, while the Alx1 proteins of more distantly related echinoderms were not. Khor and Ettensohn suggest that this is due to differences in the regions outside Domain 2. Alx1 and Alx4 are thought to be the result of an ancient gene duplication, and the acquisition of Domain 2 may have determined their different roles (Figure 1E).

This study is a sterling example of a transcription factor altering its protein sequence and presumably its affinities, leading to the functional differences of Alx1 and Alx4. Khor and Ettensohn emphasize that to fully understand these evolutionary changes more research is needed to clarify how Alx4 contributes to the formation of the skeleton in adults. We also need to examine the protein structure of Alx1 more deeply to discover what Domain 2 interacts with, and how it initiates skeleton formation.

The transcription factor Alx has the potential to become an informative model for transcription factor evolution because of its detailed gene regulatory network and the comparisons that can be made between species that diverged at different times. This will deepen our knowledge of how mechanisms beyond mutations in DNA sequences have shaped the evolution of gene regulatory networks.
